# Guideline-Based Clinical Decision Support Framework for Multimorbidity: Protocol for a Formulation and Testing Study

**DOI:** 10.2196/63339

**Published:** 2025-08-14

**Authors:** Zijun Wang, Ling Wang, Bingyi Wang, Hongfeng He, Zhewei Li, Di Zhu, Jie Zhang, Huayu Zhang, Yaolong Chen, Janne Estill

**Affiliations:** 1 Research Unit of Evidence-Based Evaluation and Guidelines Chinese Academy of Medical Sciences, School of Basic Medical Sciences Lanzhou University Lanzhou China; 2 Key Laboratory of Evidence Based Medicine of Gansu Province Lanzhou China; 3 Institute of Health Data Science Lanzhou University Lanzhou China; 4 Institute of Global Health University of Geneva Geneva Switzerland; 5 School of Population Medicine and Public Health Chinese Academy of Medical Sciences & Peking Union Medical College Beijing China; 6 School of Public Health Lanzhou University Lanzhou China

**Keywords:** multimorbidity, comorbidity, clinical decision support, protocol, burden, health care, support framework, consensus, inapplicability, monitoring

## Abstract

**Background:**

The burden of multimorbidity is increasing globally, which complicates the use of guidelines in clinical practice and health care: practitioners may need to increasingly refer to multiple guidelines with potentially conflicting recommendations.

**Objective:**

We aim to develop a guideline-based decision support framework for the management of patients with multimorbidity to help clinicians efficiently evaluate, select, and adapt recommendations focusing on the different comorbidities and aspects of multimorbidity.

**Methods:**

We will conduct the project using the following steps: (1) needs assessment (searching published literature and documents on guideline use in multimorbidity care through the study initiators, and assessing the necessity of developing a comprehensive decision-making framework focusing on multimorbidity in a broad sense), (2) establishing international working groups (a coordination team, an evidence support group, and a consensus group) by leveraging existing participants’ networks and inviting experts with relevant academic publications or activities, (3) conducting literature reviews of multimorbidity guidelines and original qualitative research involving interest-holders in multimorbidity care and/or guideline development to formulate an initial draft framework, (4) a consensus process including an expert survey and a consensus meeting, (5) formulating and releasing the final framework, and (6) testing the framework (collecting feedback through educating health professionals in different settings and applying the framework in practice to evaluate and improve it). We plan to complete the project within 3 years.

**Results:**

The project has started in March 2024 and is due to conclude in June 2026. As of May 2025, we have finished the literature reviews and qualitative studies and are currently conducting the first round of the expert survey.

**Conclusions:**

This framework will help clinicians from all levels of health care institutions to make decisions in the management of patients with multimorbidity based on the latest available evidence, and to reduce potential health risks to their patients. One limitation of this framework is that such a broad framework may not fully fit all disease combinations or realistic situations. To reduce the degree of inapplicability, after completion of the framework, we will continue to monitor its use with regular updates as needed.

**International Registered Report Identifier (IRRID):**

DERR1-10.2196/63339

## Introduction

An increasing number of people, especially older adults, are experiencing multimorbidity, a situation where the patient has multiple (chronic) conditions at the same time [1]. As a result, multimorbidity is becoming a defining challenge for health systems [2]. Interactions among drugs, interventions, and diseases can impair the patients’ quality of life and physical functioning [3,4], while health care providers also face challenges in managing the increasingly complex health situations of patients with multimorbidity [5].

Health care providers should generally refer to clinical practice guidelines or guideline-like documents such as expert consensus statements to ensure that their decisions are based on the latest evidence [6]. In multimorbidity practice, the choice of recommendations becomes, however, challenging. Due to the complex health situation of the patients, health providers may need to consult a large number of guidelines, including traditional clinical practice guidelines focusing on the target conditions and guidelines related to multimorbidity in a broader sense. Multimorbidity guidelines can further be divided into health-centered guidelines that address the condition and management of patients with multimorbidity in general, and disease-centered guidelines that address specific diseases and their combinations. Only few health-centered multimorbidity guidelines have so far been published: a systematic search conducted in 2018 identified only 8 [7]. Recommendations in the identified guidelines covered a broad spectrum of aspects of clinical and self-management, beyond the realms of traditional disease-centered guidelines [7]. Such guidelines are usually developed based on the concept of patient-centered care; however, they still face many challenges in implementation [8]. Although the use of multiple single-disease guidelines may seem to be a feasible approach, this approach does not consider the interactions or special conditions related to the coexistence of multiple diseases and may further lead to potentially hazardous side effects, overtreatment, and high costs [9]. Therefore, in the care of patients with multimorbidity, it is crucial to consider carefully which guideline recommendations are chosen and how they are applied to support clinical decision-making.

To our knowledge, no uniform standards exist yet for the use of guidelines for multimorbidity-related decision-making in clinical practice. Our project therefore aims to develop a framework to clarify the process of guideline-based multimorbidity decision-making. This framework can help clinicians evaluate, choose, and adapt recommendations from guidelines focusing on the different comorbidities and aspects of multimorbidity efficiently.

## Methods

### Overview

**Figure 1:**
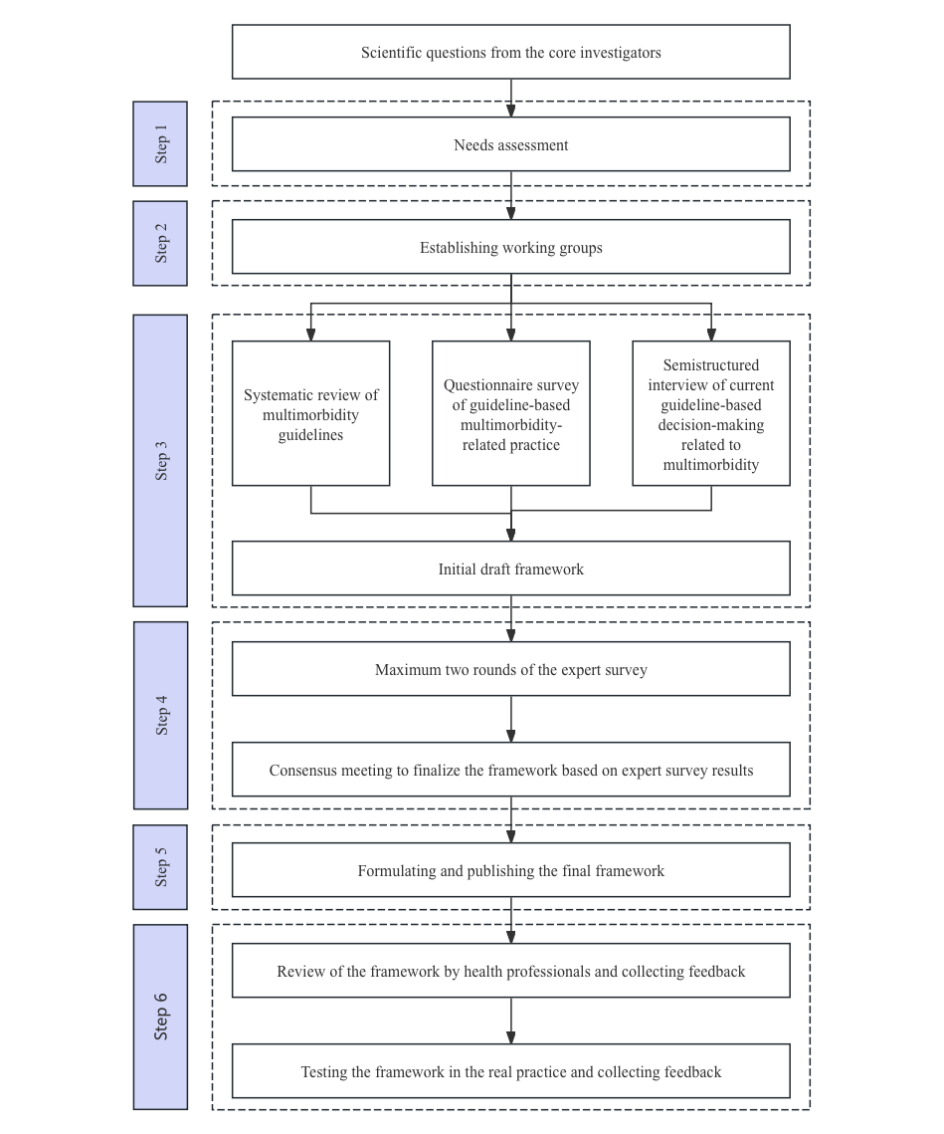
The detailed development process.

The framework we plan to develop in this project will be formed as a tree or graph. The target users include clinicians and other health care professionals at all levels of the health care system. The user will first answer questions related to the setting, patient, target condition or problems, and possibly other details depending on the final framework, which will lead to various steps to detailed instructions on how to select the recommendations from different guidelines considering also the potential interactions. We will conduct the project using methods similar to the development of reporting checklists. David Moher and colleagues from the EQUATOR (Enhancing the Quality and Transparency of Health Research) network [10] have published guidance for developing health research reporting guidelines [11]. The guidance divides the development process into initial steps, premeeting activities, face-to-face consensus meetings, postmeeting activities, and postpublication activities. The entire development can be summarized as a series of background studies and a consensus process. Based on this guidance, we plan to conduct our project with the following steps: (1) needs assessment, (2) establishing working groups, (3) formulating an initial draft framework, (4) consensus process including expert survey and consensus meeting, (5) formulating and releasing the final framework, and (6) testing the framework. The steps of the development process are illustrated in Figure 1.

### Needs Assessment

To identify the need for this new framework, we searched the published literature and documents on improving the use of guidelines in multimorbidity practice. We searched PubMed from its inception until March 2024 using terms “Multimorbidity,” “Guideline,” “Framework,” “decision support,” and their derivatives. A supplementary search in Google was also performed. We found a limited number of decision-making frameworks for multimorbidity [9,12-16], but we have various reasons to believe they are not sufficient to comprehensively guide the care of patients with multimorbidity. First, the aim of all frameworks we identified was to solve problems arising from the use of multiple single-disease guidelines, while multimorbidity guidelines (both disease- and health-centered) were usually ignored. Second, most frameworks were published at least 5 years ago without being updated thereafter. Third, many of the frameworks focused on the management of comorbidities rather than multimorbidity in general. Although these concepts are closely related, their perspectives are different. The concept of comorbidity includes the idea of focusing on a primary target disease and its interactions with other conditions, whereas multimorbidity refers to the patient’s health condition as a whole [17]. Finally, many frameworks were restricted to specific diseases or their combinations, such as type 2 diabetes and hypertension, and their scope of application is thus very narrow. Therefore, based on the current literature, there is a clear need for a comprehensive decision-making framework that focuses on multimorbidity in the broad sense.

### Establishing Working Groups

Three working groups will be established for this project: the coordination team, the evidence support group, and the consensus group.

The coordination team will consist of the 3 members who initiated the study (ZW, YC, and JE). They will lead and coordinate all steps of the project and ensure its timely completion. The coordination team is responsible for forming the initial draft framework with the help of the evidence support group, responding to the suggestions and opinions of experts during the consensus process, revising the framework, and collecting feedback through testing process of the framework. In addition, they will support the consensus group and seek advice from external parties when necessary throughout the whole process.

The evidence support group will be composed of 5-6 members with experience in evidence-based medicine and guideline methodology. This group will support the coordination team in forming the initial draft framework by (1) systematically gathering, summarizing, and/or evaluating the relevant literature and guidelines and (2) collecting the results of the questionnaire survey and interviews and performing preliminary analyses of the results.

The consensus group will be composed of 20-30 experts with experience in multimorbidity research or practice and/or guideline development or methodology. Members of the consensus group will be recruited worldwide through three pathways: (1) through contact networks of the coordination team, (2) by contacting authors of key articles relevant to the topic, and (3) by attending academic conferences related to the topic and inviting attendees to participate. Individuals who agree to participate in the consensus group will be asked to recommend additional members (the snowball method). We will continue the recruitment until at least 20 experts with sufficient diversity in terms of fields of expertise, geographical region, and other characteristics have agreed to participate. The consensus group will participate in a web-based survey and consensus meetings, provide their views during the formulation process, and approve the final version of the framework.

### Formulating the Initial Draft Framework

The coordination team will formulate the initial draft framework in 2 steps.

First, the evidence support group will collect data and ideas through a series of studies under the supervision of the coordination team (Table 1): 2 systematic reviews of guidelines, a questionnaire survey, and semistructured interviews of key experts. The 2 systematic reviews will focus on health-centered and disease-centered multimorbidity guidelines, respectively. The former review aims to include all published guidelines on how multimorbidity should be taken into consideration in health care; for the latter, we have selected diabetes, coronary heart disease, and stroke as an example, and will search all guidelines that have recommendations on any combination of these conditions. We will systematically search 6 literature databases and 9 guideline platforms for both reviews. Official websites of selected relevant societies and/or associations will also be searched for systematic reviews of disease-centered multimorbidity guidelines. Due to the expected low number of guidelines, the systematic review of health-centered multimorbidity guidelines will include all guidelines published until the date of the search. For the systematic review of disease-centered multimorbidity guidelines, we will include guidelines published in 2020 or later, which contain recommendations addressing at least 2 of the target diseases. Both reviews will collect and qualitatively analyze the recommendations and assess the methodological and reporting quality of the guidelines using the AGREE II (Appraisal of Guidelines for Research and Evaluation II) instrument and RIGHT (Reporting Items for Practice Guidelines in Health Systems) checklist, respectively. The protocols of both reviews with more details have been registered on Open Science Framework (osf.io/ymk7u and osf.io/b8ecp, respectively).

In the second step, the coordination team will discuss the findings based on their own experience to reach a consensus on the format of the framework. We will also refer to the Ariadne principle when forming the nodes of the framework. The Ariadne principle categorizes multimorbidity-related decision-making in primary care into 5 steps [19]. The Ariadne principle was formed to serve as a basis for multimorbidity management in primary care but can also give directions for multimorbidity care in general. A draft version of the framework will be built after the discussion.

**Table 1:** Summary of the studies conducted to collect the pool of key information.

Research questions	Study type	Source of information (data or participants)	Methods
What are the characteristics of current multimorbidity guidelines?	Systematic review	Health-centered multimorbidity guidelines (eg, guidelines for the care of patients with multimorbidity regardless of the diseases) Disease-centered multimorbidity guidelines (eg, guidelines for the management of hypertension in combination with other diseases)	Qualitative content analysis
How are guidelines used in multimorbidity-related practice at present?	Questionnaire survey	Individuals with experience in multimorbidity management (particularly frontline workers)	Descriptive analysis, qualitative analysis (see questionnaire in Multimedia Appendix 1)
What should we pay attention to when making multimorbidity decisions?	Semistructured interview	Individuals with experience in multimorbidity research (experts, especially guideline developers, and methodologists)	Thematic analysis [18] (see interview guide in Multimedia Appendix 2)

### Consensus Process and Formulation of the Final Checklist

We will first conduct web-based expert surveys among the consensus team, following a procedure similar to the Delphi process. During the first round of the survey, all members of the consensus group will receive a draft version of the framework through email. In this draft version, the framework will be shown as a decision tree or graph with some supplemental information about the factors that are likely to influence the choices the users make in each step. Participants will vote on 2 parts: the factors that influence the choices at each step and the rationality of the decision-making process (provided as a list of all steps from the framework). The participants will anonymously rate each factor/step on a 7-point Likert scale to indicate their level of agreement with the importance of the factor/step to be included in the framework [20]. Free-text boxes will be included in the survey to collect comments (both on the overall structure and on factors influencing each step) or propose additional factors/steps. The scale and definitions of the consensus results are shown in Table 2. The factors/steps that achieve agreement are included in the final version and removed from subsequent rounds, factors/steps with disagreement are removed from the framework (subsequent step will be modified accordingly), and factors/steps rated as ambivalent will be revised to reflect points raised by the consensus group and be included in the next round of survey. In addition, the coordination team may propose some new factors/steps based on the free-text comments. All members who participated in the first round are asked to rate the new factors/steps and factors/steps on which no consensus was reached. Free-text boxes will again be available for comments. If there are only few factors/steps that need discussion, we may consider skipping the second round and proceed directly to the consensus meeting. After a maximum of 2 rounds of survey, we will formulate a revised version of the framework, which will be discussed in the next step (consensus meeting).

We will organize a consensus meeting either on the web, in person, or in a hybrid format. All members of the coordination team and consensus group will be invited to attend and discuss the revised version of the framework. If web-based format is chosen, multiple meetings can be arranged to keep the number of attendees to a manageable level to and accommodate participants from different time zones. The meetings will be recorded and transcribed. The coordination team will review all feedback and suggestions given during the meetings and send the final version of the framework together with responses to the main suggestions to the consensus group.

**Table 2:** The 7-point Likert scale and definitions of consensus.

Score and interpretation	Definitions of conclusions regarding consensus
1=strongly disagree 2=disagree 3=somewhat disagree	“Disagreement”: more than 80% of the participants choose scores of 1 to 3
4=neither agree nor disagree (neutral) 5=somewhat agree	“Ambivalent”: the conditions for neither “agreement” nor “disagreement” are fulfilled
6=agree 7=strongly agree	“Agreement”: more than 80% of the participants choose a score of 6 or 7, and there are no major comments suggesting a revision

### Testing

We will evaluate the practical usability of the framework through 2 steps of testing. Health professionals from different types of facilities (eg, hospitals, community health centers, or nursing homes) who commonly manage patients with multimorbidity and who were not involved in any step of the development (including the surveys and interviews) will be invited to take part. In the first step, we will give an introduction of the framework to the participants and educate them how to use it. To help participants understand and try to use this framework to make decisions, we will provide a specific case (patients with diabetes and those with coronary heart disease) as an example. The participants will be asked to comment on the usability and integrity of the framework based on their experience. 

In the second step, the participants will be asked to use this framework in their daily practice related to multimorbidity management and provide feedback on the applicability and feasibility of the framework, barriers, and facilitators to this process, as well as opportunities that should be explored further to refine the framework. Feedback from these 2 steps will be collected through focus group discussions. The discussions will be recorded and analyzed using the topic analysis method.

We will compare the feedback from both steps and form suggestions and directions for future improvements to the framework. The coordination team will continue to meet twice a year to discuss any feedback from users, recent developments in multimorbidity research and policies, and the need of updating the framework or developing extensions for specific situations. Further details about the testing process are still under discussion and will be gradually determined in the future.

### Patient and Public Involvement

This study will not involve the participation of patients or the public.

### Ethical Considerations

The project has been approved by the Institute of Health Data Science Ethical Commission, Lanzhou University (reference HDS-202404-01). The framework will be disseminated through scientific articles and international conferences. All individuals who participate in different steps of the project (consensus group members, survey respondents, interviewees, and professionals involved in the testing step) will be required to provide informed consent, either in writing or via other means (such as video recording). Data from questionnaires and interviews will be treated anonymously. Participants will not receive any financial compensation.

## Results

**Figure 2:**
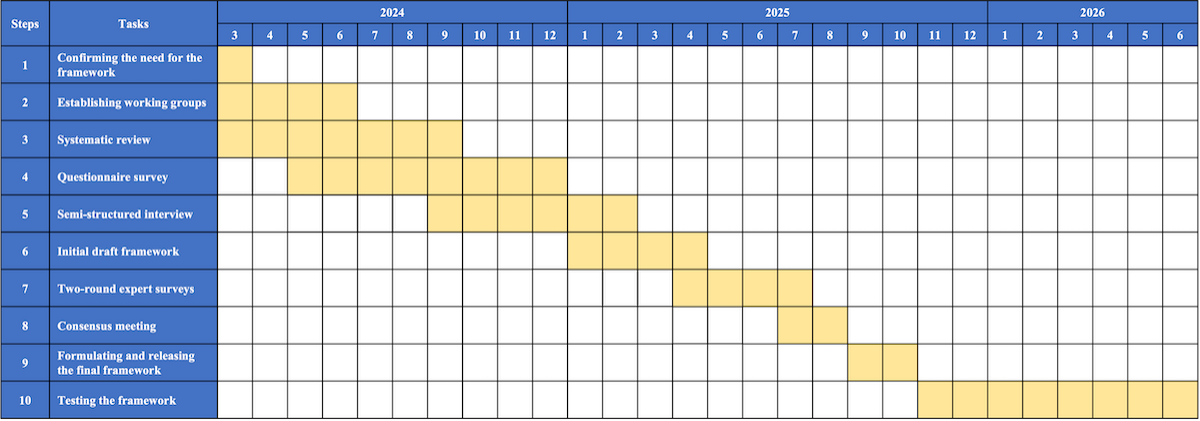
Timeline for the development of framework.

The project started in March 2024 and is due to conclude in June 2026. The proposed schedule is shown in Figure 2.

As of May 2025, we have formed all 3 working groups. The coordination team consisting of the 3 lead investigators (ZW, JE, and YC) was formed prior to the project. The evidence support group comprises 8 members with a background in evidence-based medicine. The consensus group comprises 20 experts with experience in various disciplines and from different regions of the world. In addition to the 3 groups, we have decided to invite 2 experienced clinicians specializing in multimorbidity management and guidelines as external advisors to complement the gaps in experience among the coordination team members. The coordination team and advisors will discuss the feedback from all steps of the consensus process before revising the framework.

We have, so far, finished the 2 planned systematic reviews of guidelines, a questionnaire survey, and semistructured interviews of key experts. The systematic review of health-centered multimorbidity guidelines included 19 guidelines and consensus statements and was published in November 2024 [21]. The systematic review of disease-centered multimorbidity guidelines included 82 guidelines and consensus statements with recommendations on drug therapies on combination of diabetes, coronary heart disease, and/or stroke (manuscript currently in preparation). The global survey among medical professionals with clinical experience has also been completed, with 311 valid responses (manuscript submitted for publication). Fifteen experts in multimorbidity research and guideline development with more than 10 years’ experience in the field were interviewed; the contents have been analyzed, and a manuscript is under preparation. The results with the 4 abovementioned studies were discussed and used to formulate an initial version of the framework, which consists of a decision graph with 12 nodes, explanations for each node, and for 2 nodes, also a set of factors influencing the decision to be made in the node. The first round of the consensus survey started on May 14, 2025. All members of the consensus group received a survey link including the draft version of the framework and its explanation, with a request to complete the survey in 2 weeks.

## Discussion

The main output of this project will be a framework to support guideline-based decision-making related to multimorbidity, as well as an explanatory document. This framework will help clinicians make well-informed decisions in the management of patients with multimorbidity and thus reduce potential health risks to their patients. The framework not only is limited to the selection of recommendations from disease-specific guidelines but also considers the use of general multimorbidity guidelines. The framework will cover all levels of health care from community facilities and residential homes to referral hospitals, including tailored instructions for different facility types and clinical situations. All above advantages make up for the shortcomings in the applicability and comprehensiveness of current guideline-based multimorbidity decision frameworks. The existing decision support frameworks have a very disease-specific focus and essentially only integrate recommendations from single disease guidelines [9,12-16]. These may be helpful for some decision-making in clinical practice, but they largely ignore the patient-centered model of care that is an essential part of multimorbidity management. Treating patients with multimorbidity not only consists of selecting the optimal therapy based on the clinical situation but also requires considering factors like the patient’s preferences and values, practical issues related to the social situation and daily activities, and the quality of life in general [22-24]. Health-centered multimorbidity guidelines focus on these issues, but these guidelines are usually not addressed by the existing tools, and clinicians may not be aware of the existence of such recommendations. In contrast, our framework will cover all aspects of patient care, including also the challenges related to multimorbidity itself.

There are several potential limitations to our framework. First, multimorbidity is a highly complex issue, and management policies and the availability of applicable guidelines vary across countries, regions, and health care settings. This diversity makes it challenging to fit such a broad topic into a simplified decision tree. As a result, our framework may have limited applicability in certain contexts. Second, implementing the framework in clinical decision-making requires specific resources and expertise. For instance, during the development the framework, we may assume that all relevant guidelines are readily available. However, in practice, access to these guidelines may be restricted due to differences in hospital levels or institutional policies. Third, a persistent challenge in multimorbidity research and clinical practice is the lack of strong direct evidence. Consequently, even if the framework is implemented, final decisions may still rely on expert judgment rather than strong recommendations based on high-quality evidence. Fourth, shifting clinical attitudes, particularly among specialists, may be difficult. While we are committed to refining the framework for practical use, we cannot guarantee its seamless integration into clinical practice. We can mitigate the influence of these limitations by continuously revising the framework based on feedback from users. With the help of partners across the world, we will adapt the framework to the local contexts of different settings and consider developing extension versions for specific situations where the core framework alone is not sufficient. Ultimately, we hope this framework will lead to a more standardized approach in the care of patients with multimorbidity in different situations, guiding the way toward more personalized and patient-centered health care.

## Appendix

Multimedia Appendix 1 Questionnaire.

Multimedia Appendix 2 Interview guide.

## Abbreviations

AGREE II: Appraisal of Guidelines for Research and Evaluation II
EQUATOR: Enhancing the Quality and Transparency of Health Research
RIGHT: Reporting Items for Practice Guidelines in Health Systems
