# Supplementary material for: Guideline-Based Clinical Decision Support Framework for Multimorbidity: Protocol for a Formulation and Testing Study
Source: JMIR Res Protoc. 2025 Aug 14;14:e63339. doi: 10.2196/63339 (PMC12395098; doi:10.2196/63339)
Supplement: Multimedia Appendix 1 [file resprot_v14i1e63339_app1.docx]

**Supplementary file 1: Questionnaire**

**Survey of the use of, and adherence to, guidelines in multimorbidity-related practice**

**Introduction**

Dear Participant,

Thank you for taking the time to participate in this survey. Estimated time to complete this questionnaire is 10 minutes.

Multimorbidity (a co-existence of two or more chronic conditions) is a growing global challenge with substantial effects on individuals, healthcare systems, and the entire society. Guidelines and expert consensus statements are tools that guide the daily practice of clinicians and other healthcare providers. The management of multimorbid patients involves often several different guidelines focusing on the different conditions and aspects of care. This survey aims to explore the use of, and

adherence to, guidelines in multimorbidity-related practice.

The survey is a part of a PhD project aiming to formulate a support framework for guideline-based decision making in clinical practice involving multimorbid patients. The results of the survey will be used as one of the supporting materials to inform the development of this framework. The project is funded by China National Academy of Medical Sciences (2021RU017).

Your privacy is of utmost importance to us, and all responses will be kept strictly confidential and used for research purposes only. The results will be published anonymously as a scientific article. This study has been approved by the Institute of Health Data Science, Lanzhou University (reference no. HDS-202404-01).

If you have any questions, please contact the coordinator, Ms. Zijun Wang ([bdwzj_0312@163.com](mailto:bdwzj_0312@163.com)).

* 1. I confirm that:

- I am currently, or have been in the past, actively and directly involved in the care of patients or people with special needs as a physician, nurse, caregiver, pharmacists, or other health professional

- I have read the information above and agree to participate in the survey.

□Yes, I confirm

**Glossary**

**In order to ensure that the questionnaire is fully understandable, we have explained some relevant terms below. Please read the glossary carefully before starting the survey.**

**Multimorbidity**: co-existence of two or more chronic conditions.

**Comorbidity**: other (chronic) health conditions of a multimorbid patient than the primary target condition.

**Guideline**: Clinical practice or public health guideline, expert consensus statement, or other similar document that includes recommendations intended to inform clinical or other health practice.

**Health centered multimorbidity guideline**: Guideline with recommendations that focus on the patient's overall health rather than any specific disease(s). Example: the guideline Multimorbidity: clinical assessment and management published by NICE (https://www.nice.org.uk/guidance/ng56)

**Disease centered multimorbidity guideline**: Guideline with recommendations that focus on the management of specific disease combination, like “guidelines for hypertension with diabetes” (for example, https://diabetesjournals.org/care/article/40/9/1273/36772/Diabetes-and-Hypertension-A-Position-Statement-by)

**Single disease guideline**: Guideline with recommendations that focus on only one disease, like “guidelines for hypertension” (for example, https://www.who.int/publications/i/item/9789240033986). These guidelines may also include recomendations that consider the impact of, or interaction with, comorbidities, but this is not necessarily the case.

**Basic information of the respondent**

2. Name

First name_______

Last name_______

3. Email

Email address_______

4. Affiliation

_____________________

* 5. Gender

□Male

□Female

□Others

□Prefer not to disclose

* 6. Country

_____________________

* 7. Occupation/role

□Clinician

□Nurse

□Pharmacist

□Researcher

□Administrative personnel

□Other (please specify) _________

* 8. Type of facility

□Hospital

□Health center/clinic

□Nursing home

□Research institution

□Governmental agency

□Other (please specify) _________

* 9. Professional title or position

□Senior consultant/leadership position

□Registrar/intermediate position

□Junior position

□Intern/student _________

* 10. Years on duty

_____________________

* 11. Conflict of interest

□I have no conflict of interest to declare

□I have some conflict of interests related to this project (please specify) _________

**Knowledge and experience with multimorbidity**

* 12. Are you familiar with the concept of multimorbidity?

□Very familiar

□Familiar

□I know the concept but am not very familiar with it

□I did not know the concept before participating in this survey

* 13. How often do you work with multimorbid patients/clients or their management?

□Most of my patients/clients are multimorbid

□Multimorbidity is common among my patients/clients

□Sometimes but not very often

□Never

* 14. Do you take multimorbidity and different comorbidities of patients into account in your daily practice in some particular way?

□Yes, I always pay attention on the comorbidities

□Sometimes depending on the situation

□Rarely, I usually just focus on the primary target condition

**Use of guidelines in multimorbidity practice**

* 15. Do you use guidelines when treating or managing patients with multimorbidity?

□Always

□Sometimes

□Never

* 16. Which kind of guidelines have you used in your practice?

□Health centered multimorbidity guidelines

□Disease centered multimorbidity guidelines

□Multiple single disease guidelines for different comorbidities together

□Single disease guidelines for the primary target condition only

* 17. Do you use any decision support framework or tool to select guidelines or recommendations when managing multimorbid patients?

□No

□Yes (please specify) _________

* 18. Reasons you prefer to not use health centered multimorbidity guidelines include:

Health centered multimorbidity guideline: Guideline with recommendations that focus on the patient's overall health rather than any specific disease(s).

□Guidelines are not available

□Guidelines are not applicable

□Guidelines are not reliable

□Other reasons (please specify) _________

□Not applicable

* 19. Reasons you prefer to not use disease centered multimorbidity guidelines include:

Disease centered multimorbidity guideline: Guideline with recommendations that focus on the management of specific disease combination, like “guidelines for hypertension with diabetes”

□Guidelines are not available

□Guidelines are not applicable

□Guidelines are not reliable

□Other reasons (please specify) _________

□Not applicable

* 20. Reasons you prefer to not use multiple single disease guidelines include:

Single disease guideline: Guideline with recommendations that focus on only one disease, like “guidelines for hypertension”. These guidelines may also include recommendations that consider the impact of, or interaction with, comorbidities, but this is not necessarily the case.

□Guidelines are not available

□Guidelines are not applicable

□Guidelines are not reliable

□There are too many guidelines and it is difficult to choose between them

□Contradictions between guidelines

□Following multiple recommendations can lead to overtreatment

□Following multiple recommendations can lead to high costs

□Following multiple recommendations can lead to too complex care services

□Interactions of different conditions or their treatments unclear

□Following multiple recommendations can lead to work pressure

□It is not my duty to manage comorbidities, I only need to focus one target condition

□Other reasons (please specify) _________

□Not applicable

* 21. Reasons you prefer not to use single disease guidelines in general include:

Single disease guideline: Guideline with recommendations that focus on only one disease, like “guidelines for hypertension”. These guidelines may also include recommendations that consider the impact of, or interaction with, comorbidities, but this is not necessarily the case.

□Guidelines are not available

□Guidelines are not applicable

□Guidelines are not reliable

□There are too many guidelines and it is difficult to choose between them

□Interactions of different conditions or their treatments unclear

□Not sufficient information about risks associated with comorbidities

□Other reasons (please specify) _________

□Not applicable

* 22. Reasons you have used existing guidelines although they are not fully applicable or reliable include:

□Policy requirement

□No better choice

□Used together with other sources of evidence

□Other reasons (please specify) _________

□I never use guidelines that are not applicable

* 23. Do you think the current guidelines are sufficient to guide your daily practice?

□Yes

□No (please explain why) _________

* 24. What actions do you take if the existing guidelines do not meet the needs or if there are no guidelines?

□Refer to systematic reviews and primary studies such as randomized controlled trials

□Consult experts

□Rely on your own experience

□Other actions (please specify)

* 25. What do you think is needed to improve multimorbidity practice? Please rank the following suggestions in order of importance

□Develop a tool or methodology to guide the use of multiple guidelines

□Develop more multimorbidity guidelines

□Address multimorbidity better in disease guidelines

26. If you have more suggestions to improve multimorbidity practice, please write the suggestions and their rank here

__________________
