# Supplementary material for: Guideline-Based Clinical Decision Support Framework for Multimorbidity: Protocol for a Formulation and Testing Study
Source: JMIR Res Protoc. 2025 Aug 14;14:e63339. doi: 10.2196/63339 (PMC12395098; doi:10.2196/63339)
Supplement: Multimedia Appendix 2 [file resprot_v14i1e63339_app2.docx]

**Supplementary file 2: Interview guide**

**Interview on Multimorbidity Guidelines and Clinical Decision Making**

Dear Participant, thank you for taking the time to participate in this interview. Before we begin the interview, please allow us to give you a brief overview of the background and process of the interview.

**Background**

Multimorbidity (a co-existence of two or more chronic conditions) is a growing global challenge with substantial effects on individuals, healthcare systems, and the entire society. Guidelines and expert consensus statements are tools that guide the daily practice of clinicians and other healthcare providers. However, the use of guidelines in multimorbidity practice can be fraught with problems, as the management of multimorbid patients often involves several different guidelines focusing on different conditions and aspects of care. Decisions following multiple guidelines can lead to potential disease and drug interactions that affect the patient's health. To avoid or reduce this risk, our team plans to develop a guideline-based decision support framework for clinical practice and management of multimorbid patients. This interview aims to collect opinions on what should be considered when using guidelines to treat or manage multimorbid patients. The results of the interview will be used as one of the supporting materials to inform the development of the guideline-based decision support framework.

Before the interview, we recommend you to familiarize yourself with the questions and the attached documents.

**Interview process**

1. Our team will conduct an interview with you in person or online, lasting approximately 0.5 to 1 hour, which will be audio-recorded and transcribed verbatim.
2. Before the interview begins, we will ask you to give your informed consent and sign an informed consent form.
3. If you feel that any question during the interview is sensitive or makes you feel uncomfortable, you can refuse to answer.
4. We will send the transcript of the interview to you for review within 2 weeks after the interview. Minor revisions will be accepted if they do not contradict with the meaning of the original interview.
5. Your privacy is of utmost importance to us, and all responses will be kept strictly confidential and used for research purposes only. The results will be published anonymously as a scientific article.

If you have any questions, please contact the coordinator, Ms. Zijun Wang (bdwzj_0312@163.com).

**Interview on Multimorbidity Guidelines and Clinical Decision Making**

**Statement of Informed Consent:**

1. I have been informed of the purpose of the study, the person in charge, the source of funding, the content of the information collection, and the confidentiality measures for this interview.
2. I have also been informed that I can contact the study leader by email when I have questions, want to reflect difficulties, concerns, or suggestions for the study, want to obtain further information, or offer help with the study.
3. I understand that I may voluntarily choose to participate or not participate in this study, and that I may withdraw from this study at any time after the start of the interview without any reason.
4. I have read this informed consent form and agree to participate in this study. I will be given a copy of this informed consent form that includes my signature and the signature of the interviewer.

**Signature of interviewee_________ Date of signature _________**

**Interviewer Statement:**

I confirm that I have explained the details of this study to the interviewee, including the purpose of the study, the person in charge, the source of funding, the content of the information collection, and the measures of confidentiality.

**Signature of interviewer _________ Date of signature _________**

**Please read carefully the following definitions that we will use frequently in the interview:**

**Health centered multimorbidity guideline:** Guideline with recommendations that focus on the patient's overall health rather than any specific disease(s). Example: the guideline Multimorbidity: clinical assessment and management published by NICE (https://www.nice.org.uk/guidance/ng56)

**Disease centered multimorbidity guideline:** Guideline with recommendations that focus on the management of specific disease combinations, like “guidelines for hypertension with diabetes” (for example, https://diabetesjournals.org/care/article/40/9/1273/36772/Diabetes-and-Hypertension-A-Position-Statement-by)

**Single disease guideline:** Guideline with recommendations that focus on only one disease, like “guidelines for hypertension” (for example, https://www.who.int/publications/i/item/9789240033986). These guidelines may or may not also include recommendations that consider the impact of, or interaction with, comorbidities.

**If you have any questions related to these terms and their meaning, please ask the investigator before starting the interview.**

**Content of the interview:**

1. Please briefly explain your role and experience on multimorbidity research and guideline research.
2. In your experience, how are guidelines currently applied in multimorbidity related clinical and healthcare decision-making?
3. What are the facilitators and barriers that in your opinion influence guideline-based multimorbidity decision making?
4. Considering the facilitators and barriers mentioned above, as well as your own experience, how do you think the use of guidelines could be promoted in multimorbidity related decision-making?
5. When using guidelines to guide multimorbidity-related clinical decision-making, what essential issues in your opinion cannot be resolved with the existing methods?
6. We would like to develop a tool or framework to guide clinicians in the appropriate use of guidelines in decision-making in the management of multimorbid patients. What do you think this tool could look like?
7. Is there any other information you would like to share with us?
